# Sad benefit in face working memory: An emotional bias of melancholic depression

**DOI:** 10.1016/j.jad.2011.08.002

**Published:** 2011-12

**Authors:** Stefanie C. Linden, Margaret C. Jackson, Leena Subramanian, David Healy, David E.J. Linden

**Affiliations:** a Institute of Psychiatry, Kings College, London, UK; b Wolfson Centre for Clinical and Cognitive Neuroscience, School of Psychology, Bangor University, Bangor, UK; c Dept. of Psychological Medicine & Neurology, Cardiff University, Cardiff, UK

**Keywords:** Depression, Melancholia, Working memory, Facial expression, Emotion

## Abstract

Emotion biases feature prominently in cognitive theories of depression and are a focus of psychological interventions. However, there is presently no stable neurocognitive marker of altered emotion–cognition interactions in depression. One reason may be the heterogeneity of major depressive disorder. Our aim in the present study was to find an emotional bias that differentiates patients with melancholic depression from controls, and patients with melancholic from those with non-melancholic depression. We used a working memory paradigm for emotional faces, where two faces with angry, happy, neutral, sad or fearful expression had to be retained over one second. Twenty patients with melancholic depression, 20 age-, education- and gender-matched control participants and 20 patients with non-melancholic depression participated in the study. We analysed performance on the working memory task using signal detection measures. We found an interaction between group and emotion on working memory performance that was driven by the higher performance for sad faces compared to other categories in the melancholic group. We computed a measure of “sad benefit”, which distinguished melancholic and non-melancholic patients with good sensitivity and specificity. However, replication studies and formal discriminant analysis will be needed in order to assess whether emotion bias in working memory may become a useful diagnostic tool to distinguish these two syndromes.

## 1 Introduction

A bias for negative information is central to cognitive theories of depression (Beck, 1976; Mathews and MacLeod, 2005). Patients with depression have shown selective attention to sad but not angry or happy faces on a dot probe task (Gotlib et al., 2004) and mood-congruent (that is, negative) evaluation of neutral or ambiguous material (Gotlib and Joormann, 2010). A similar preference for negative information was observed in long-term memory studies in patients with depression or dysphoric individuals (Gilboa-Schechtman et al., 2002; Jermann et al., 2008; Ridout et al., 2003, 2009), most consistently in those employing free recall (Gotlib and Joormann, 2010). Such biases are not confined to the visual modality but have also been demonstrated for emotional prosody (Péron et al., 2011).

However, not all studies show emotion biases, let alone performance improvements for negative material in depression. The evidence is most consistent for tasks where patients have to suppress memory traces of negative material such as negative priming (Goeleven et al., 2006; Joormann, 2004) or directed forgetting of self-referential words (Power et al., 2000), where socially relevant stimuli such as faces are used, and where presentation times are relatively long (1 s or more) (De Raedt and Koster, 2010). Moreover, most previous studies on emotion-specific effects on attention and memory have shown selective interference and disengagement, rather than performance enhancements (De Raedt and Koster, 2010).

Only a few studies have specifically probed emotional working memory biases in depression. This is surprising because the active maintenance of mood-congruent material in working memory would constitute a particularly pervasive cognitive bias because of the wide range of functions that depend on working memory. In one study patients (but not healthy individuals undergoing sad mood induction) took longer to reject intrusions from irrelevant word lists if they contained negative material (Joormann and Gotlib, 2008). The authors suggested that this indicates the reduced ability to remove previously relevant negative material from working memory when it has become irrelevant and that this dysfunctional cognitive style might sustain ruminations. Global working memory impairment, reported in some studies (Rose and Ebmeier, 2006), does not seem to account for emotion biases in depression (Gotlib and Joormann, 2010).

The absence of a clear sad benefit in dysphoria in other previous work (Noreen and Ridout, 2010) may be explained by the characteristics of the sample of that study, which included only healthy individuals who scored high on a depression scale. Deveney and Deldin (2004) did investigate patients with Major Depressive Disorder (MDD) and did not find a bias for sad faces in working memory, but they used a load of only one face. Face working memory performance at load 1 may be close to ceiling for all categories (Jackson and Raymond, 2008). Our previous work has shown that emotion effects on face WM start to emerge only at a load of two faces (Jackson et al., 2009). In the present study we therefore used a working memory protocol where participants had to retain two faces, which has shown good sensitivity to detect emotion biases in patient groups (Linden et al., 2010; Subramanian et al., 2010).

Another reason that previous work on cognitive biases has not yielded stable neurocognitive markers of depression could be the heterogeneity of the depressive syndrome. In particular, most studies did not adopt the clinically important distinction between melancholic and non-melancholic depression. It is often argued that melancholia is a severe form of depression on a continuum of depressive illness. However, the concept of melancholia as a distinct clinical entity has recently received considerable support (Parker et al., 2010; Taylor and Fink, 2008). This separation of melancholia from other depressive subtypes, which we adopt here, is based on its distinct clinical features (psychomotor disturbances, quality of mood, lack of mood reactivity, vegetative signs) and different treatment responses (Hildebrandt et al., 2003; Parker et al., 2010; Perry, 1996). The diagnostic significance of psychomotor disturbances as a distinguishing feature between melancholic and other subtypes of depression has been supported by several studies (Parker, 2007; Schrijvers et al., 2008). The DSM-IV (Diagnostic and Statistical Manual of Mental Disorders) allows a specification of melancholic subtype of depression defined by clinical symptoms and signs. It is widely recognised that the depressive syndrome in its current definition is in many respects too broad, and that clinical and neurocognitive subtyping is needed. This study was intended to contribute to the identification of a potential cognitive marker for depression, or specifically for melancholia, through a negative emotional bias in working memory.

## 2 Method

### 2.1 Participants and diagnostic assessment

20 outpatients with melancholic depression, 20 healthy age-, gender- and handedness-matched controls, and 20 outpatients with non-melancholic depression participated in the study (Table 1). All participants gave written informed consent after full explanation of the study procedures. The study was approved by the ethics committee of the School of Psychology, Bangor University, and the Local Research Ethics Committee of the National Health Service. The control participants, who were recruited through the University's community panel, reported no history of neurological or psychiatric disorder or illicit drug use. Authors S.L. and D.H., board-certified psychiatrists, confirmed the clinical diagnosis of depression according to DSM-IV-TR-criteria (American Psychiatric Association) with the Structured Clinical Interview for DSM-IV (SCID) (First et al., 2002). We only included patients aged between 18 and 65 with MDD, excluding patients with a Major Depressive Episode in Bipolar Disorder. We further excluded patients with comorbid other psychiatric, neurological or substance-related disorders. The severity of depressive symptoms was assessed with the 21-item Hamilton Rating Scale for Depression (HDRS) (Hamilton, 1980).

The differentiation into melancholic and non-melancholic depression was based on the criteria for the DSM Melancholic Features Specifier and was made by the same two psychiatrists, who were initially blind to the outcome of the experiments. However, our criteria were stricter than those of the DSM-IV manual, where one A criterion and three B criteria are required to make the diagnosis “with melancholic features”. In order to meet the inclusion criteria for our study, patients had to fullfill all eight criteria (2 A criteria and 6 B criteria, see Table 2). We reasoned that such strict criteria would result in the identification of purer melancholic syndromes and enhance the chance of identifying neurocognitive markers in a relatively small sample. The criteria were applied to the most severe period of the current MDD episode. None of the patients in our non-melancholic group met the broader DSM-IV criteria for a “with melancholic features” diagnosis.

We assessed pre-morbid verbal intelligence with the National Adult Reading (NART) test (Russell et al., 2000). We ruled out deficits in face recognition with the Benton test (Benton et al., 1983). In this test participants match target faces to simultaneously presented test faces. The test is administered in a booklet and there is no time constraint. All participants had normal or corrected to normal vision.

### 2.2 Procedure

#### 2.2.1 Experiment 1: working memory for emotional faces

**Fig. 1:**
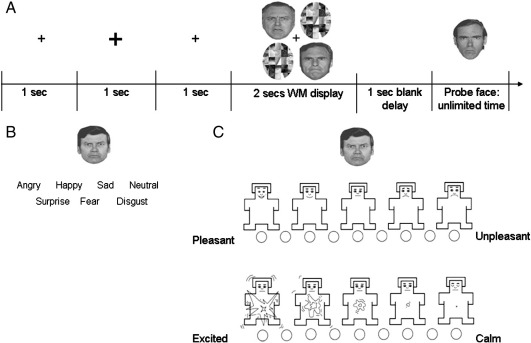
The emotional face paradigms. (A) Working memory. Participants viewed two faces (expressing the same emotion, angry, happy, sad, fearful or neutral) for 2 s. The 4 position array was chosen because of compatibility with other work probing load effects on emotional working memory (Jackson et al., 2009) and with our previous work in patients with Parkinson's disease (Subramanian et al., 2010) and schizophrenia (Linden et al., 2010). After a 1 s interval, a probe face with the same emotional expression was presented and participants had to judge whether it matched one of the previous faces in the encoding display. (B) Expression classification task for the basic emotions. Participants categorised emotions with the appropriate labels. (C) Arousal/valence ratings. Participants rated how aroused and pleasant each face made them feel using the self-assessment manikin.

We assessed working memory for emotional faces with a modified version of the paradigm reported in Linden et al. (2010). In this task participants were oriented to the centre of the computer screen by a small fixation cross presented for 1000 ms (Fig. 1). At the start of each trial, the fixation cross increased in size for 1000 ms after which it returned to its original size for another 1000 ms. Then two faces and two scrambled faces were presented for 2000 ms in a 2 × 2 encoding array that subtended 2.86° visual angle in width and 2.96° in height. After a 1000 ms blank retention interval a single face was presented in the centre of the screen and participants were asked to decide whether this face had been present in the previous display by pressing either yes or no on the keyboard.

All faces in any one trial displayed the same emotion, thus there was no competition between emotions for WM resources. The task involved an identity decision (“was this person present or not?”) and emotional expression was irrelevant to the task. Responses were not speeded and no feedback was provided, and participants were free to disengage fixation. We did not include a verbal suppression task because patients could not cope with this extra cognitive load when engaging with the task.

Stimuli were displayed on a 13-inch Toshiba laptop monitor (32 bit true colour, resolution 1024 × 768 pixels), generated by E-Prime software (Version 1.1; Schneider et al., 2002). Greyscale face images of six adult males each expressing five emotions (angry, happy, neutral, sad, fearful) were used. The faces were taken from the Ekman and Friesen collection (Ekman and Friesen, 1976). 16 trials per emotion were presented in a pseudo-random order in four blocks.

#### 2.2.2 Experiment 2: emotion classification

We investigated the ability to classify facial emotions using the same six male faces from the Ekman set with seven different emotional expressions (happy, angry, neutral, sad, disgusted, fearful, surprised). E-Prime software was used and faces were displayed on a 13-inch Toshiba laptop monitor. The experiment consisted of 42 trials (each of the 6 male faces in 7 different emotions shown once). In each trial a face with an emotional expression that subtended 3.6° visual angle in width and 4.5° in height, appeared along with the seven written emotion labels printed below it. The participant's task was to identify the expression displayed on the face by clicking the correct emotion label with the computer mouse.

#### 2.2.3 Experiment 3: arousal and valence ratings

We investigated participants' arousal and valence judgements with a computerised version of the self-assessment manikin (Bradley and Lang, 1994). Participants rated the arousal (calm = nonaroused to exciting = aroused) and valence (pleasant = positive to unpleasant = negative) associated with the face on a nine point scale. Only the five emotions used in experiment 1 and items correctly categorised in experiment 2 by the participant were included in the valence and arousal ratings.

### 2.3 Participant matching

We matched the control group to the melancholic patients for age, handedness and gender. There was no difference between any of the groups in premorbid intelligence as measured by the NART (melancholic patients: 111.10 [10.79], controls: 113.85 [9.70]; non-melancholic patients: 113.05 [8.98], *F*(2,57) = 0.413, *p* = .663). There was a trend in the 1-way ANOVA to a main effect for age, which was driven by lower age in the non-melancholic patient group, *F*(2,57) = 2.343, *p* = .105. Chi square test did not reveal significant group differences in gender or handedness (all *p*s > .2).

### 2.4 Data analysis

D prime (d′) scores on the working memory task, percent correct on the categorisation task and arousal and valence ratings were our dependent measures. D′ scores are computed as the difference between z-transformed hits and false alarms and represent the standard performance measure for change detection tasks (Green and Swets, 1966). We analysed the dependent measures in separate repeated measures analyses of variance (ANOVAs) with the between-subject factor “group” (three levels: controls, melancholic patients, non-melancholic patients) and the within-subject factor “emotion” (five levels for the WM task and the arousal and valence ratings; seven levels for the classification task). P-values were Greenhouse–Geisser corrected where the assumption of sphericity was violated. We followed up the main effects and interactions with post-hoc one-way ANOVAs, Scheffe or t-tests, as appropriate. We also correlated the size of the sad benefit (performance difference between sad and the average of all other faces: d′_sad_ − (d′_angry_ + d′_happy_ + d′_neutral_ + d′_fearful_) / 4) with the HDRS scores and duration of illness.

## 3 Results

### 3.1 Working memory (experiment 1)

**Fig. 2:**
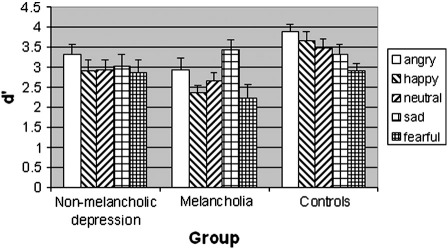
Performance of patients with non-melancholic depression, melancholia and healthy control participants on the working memory task for angry, happy, neutral, sad and fearful faces, measured in d′ values (y-axis) and their standard errors of the mean.

The ANOVA of the d′ values of the working memory task (Table 3) showed main effects of emotion, *F*(4,228) = 5.208, *p* < .001, and group, *F*(2,57) = 4.995, *p* = .01, and an interaction between emotion and group, *F*(8,228) = 2.074, *p* = .039. Separate one-way ANOVAs for each group revealed main effects of emotion in the melancholic patients, *F*(4,76) = 4.773, *p* = .002 and controls, *F*(4,76) = 4.629, *p* = .002, but not in the non-melancholic patients, *F*(4,76) = 0.653, *p* = .63. Pairwise comparisons revealed significantly higher performance for sad compared to happy, *t*(19) = 5.970, *p* < .001, 2-tailed, fearful, *t*(19) = 3.261, *p* = .004, and neutral, *t*(19) = 2.563, *p* = .019, faces, and a trend to higher performance for sad compared to angry faces, *t*(19) = 1.774, *p* = .092, in the melancholic group. Of these, the contrasts sad vs. happy and sad vs. fearful survive Bonferroni correction for 10 comparisons. There was no peak for sad faces in any of the other groups (Fig. 2). In the control group, the significant (surviving Bonferroni correction) pairwise comparisons were angry vs. fearful, *t*(19) = 4.288, *p* < .001, happy vs. fearful, *t*(19) = 3.954, *p* = .001, and neutral vs. fearful, *t*(19) = 3.566, *p* = .002. Thus, the interaction between the factors group and emotion was driven by the different direction of emotion effects in the melancholic (better memory for sad faces) and control (worse memory for fearful faces) groups.

The performance difference between sad and the average of all other faces (d′_sad_ − (d′_angry_ + d′_happy_ + d′_neutral_ + d′_fearful_) / 4) (“sad benefit”) differentiated between patients with melancholic depression and controls, and between patients with melancholic and non-melancholic depression with 80% sensitivity and 80% specificity. The cutoff point of the d′ difference was 0.5.

### 3.2 Emotion classification (experiment 2)

The ANOVA of the accuracy scores (Table 4) on the emotion classification task showed a main effect of emotion, *F*(4.454,253.869) = 19.993, *p* < .001, but no interaction between emotion and group, *F*(8.908,253.869) = 1.216, *p* = .286, and no group effect, *F*(2,57) = 2.090, *p* = .133. The main effect of emotion was driven by better classification accuracy for happy faces compared to all other conditions (all *p*s < .001).

### 3.3 Arousal and valence ratings (experiment 3)

Patients and controls showed no major differences on arousal and valence ratings (Tables 5 and 6). The ANOVA of the arousal data showed a main effect of emotion, *F*(3.180,162.165) = 17.540, *p* < .001. Neither the main effect for group nor the interaction between the two factors was significant. Arousal ratings were significantly higher for angry and fearful faces compared to all other conditions (all *p*s < .001) (but did not differ between angry and fearful faces) and for sad, *p* < .001, and happy, *p* = .028, compared to neutral faces.

The ANOVA of the valence data showed a main effect of emotion, *F*(1.844,94.041) = 89.347, *p* < .001, but no significant main effect of group and no interaction. The main effect of emotion was driven by higher valence for happy compared to all other faces (all *p*s < .001), lower valence of angry, fearful and sad than neutral faces (all *p*s < .001) and lower valence of angry, *p* < .001, and fearful, *p* = .004, than sad faces.

### 3.4 Performance correlation with symptoms

Spearman correlation between sad benefit in the melancholic group and HDRS scores revealed no significant association, rho = −.006, *p* = .971, 2-tailed. Likewise, Pearson correlation between the sad benefit and duration of illness showed no significant association, r = −.136, *p* = .404.

## 4 Discussion

Our finding of a sad benefit in face working memory supports the cognitive theories of melancholic depression that posit a bias for negative, particularly sad information. However, such biases can also be obtained through negative mood induction in healthy individuals (Bower, 1981; Bradley et al., 1997; Ridout et al., 2009), especially in at-risk individuals (Joormann et al., 2007), which has raised the question whether the negative emotion bias is secondary to the mood change. Yet negative biases may also persist beyond depressive episodes and thus constitute a vulnerability marker to rather than a consequence of the illness (Fritzsche et al., 2010; Gotlib and Joormann, 2010; Joormann and Gotlib, 2007). The lack of correlation of the “sad benefit” with symptom severity or duration of illness in our study suggests that it is not a consequence of the mood change but might rather be a primary neurocognitive feature of melancholic depression. Although cognitive bias theories have greatly influenced current thinking on mood disorders and been applied productively in the development of cognitive behavioural therapies, the question of causality has remained open (Mathews and MacLeod, 2005). Findings of cognitive biases that are independent of severity of symptoms, as in the present study, lend some support to a causal (rather than reactive) role of cognitive biases in the development of depression. However, ultimately this question will have to be addressed by longitudinal studies in high-risk individuals. The present paradigm will be useful for studies that address the question whether cognitive biases predict the development of depression, and whether their remediation helps with symptom improvement.

Melancholic and non-melancholic depression are distinct clinical syndromes and may dissociate on some biological measures (Gold and Chrousos, 2002; Parker et al., 2010; Power et al., 2000). However, most neurocognitive studies of depression have not investigated these syndromes separately. Our results provide strong evidence that different cognitive processes underlie these two syndromes because patients with non-melancholic depression essentially showed the same pattern as controls (albeit with descriptively slightly impaired performance), whereas patients with melancholic depression showed an altered emotion bias both compared to controls and non-melancholic patients. These neurocognitive differences between melancholia and non-melancholic depression may lend further support to the inclusion of melancholia as a separate entity in the DSM-V.

In a recent study with a similar paradigm in patients with Parkinson's disease (PD) we found increased performance for sad faces in the hypodopaminergic state, which reverted to the “normal” preference for angry faces after dopaminergic treatment (Subramanian et al., 2010). The monoamine systems show multiple interactions with the HPA axis, and lack of dopamine has been postulated particularly for melancholic depression and psychomotor symptoms (Martinot et al., 2001; Schrijvers et al., 2008). Dopaminergic agents have antidepressant properties both in PD (Yamamoto and Schapira, 2008) and depression (Dhillon et al., 2008). One tentative explanation of the negative cognitive bias in melancholic depression may thus be that it reflects an underlying dopaminergic deficit. This needs to be tested further in intervention or radioligand studies. Failure to differentiate according to clinical subsyndromes may be one of the reasons why radioligand studies have so far failed to provide conclusive support for monoaminergic deficit models of depression (Nikolaus et al., 2009).

Because only patients who were experiencing a depressive episode were included we cannot ascertain whether the sad benefit in WM persists during remission and may be an enduring emotional bias contributing to vulnerability to relapse. Furthermore, in the absence of direct evidence from neuroimaging or effects of specific pharmacological interventions in patients with depression, our interpretations of the sad benefit as indicator of dopaminergic hypofunction is presently still speculative. Another limitation is that our melancholic patients were more severely depressed, according to HDRS scores, than the non-melancholic patients. However, this difference is unlikely to have accounted for the qualitative difference in emotion biases, and we found no significant correlation between the sad benefit and symptom severity.

The identification of patients with melancholic depression requires an in-depth interview, a careful clinical history and often observation over an extended time period. An initial neurocognitive screening tool could therefore be of clinical use for early identification. The successful classification of melancholia patients vs. controls and vs. non-melancholic patients with 80% sensitivity and specificity is therefore of potential clinical interest, but formal testing of its sensitivity and specificity in distinguishing melancholic from non-melancholic depression through discriminant analysis in an independent sample would be required. If the sad bias is indeed a marker of lack of dopamine, it should revert after treatment with dopaminergic (and possibly other catecholaminergic) agents, thus providing a neurocognitive treatment marker. Our finding would thus respond to the widely recognised need for additional outcome measures that are independent of self-report.

Finally psychological interventions that target dysfunctional cognitive biases and metacognitive beliefs such as Cognitive Behavioural Therapy (CBT) could specifically focus on patients' bias for sad material. In addition to explicit strategies, implicit procedures using operant conditioning or distractor devaluation may be possible. Moreover, changing the sad bias in working memory and other cognitive domains may become a new outcome measure not only for pharmacological treatment but for psychological interventions as well.

## Role of the funding source

This work was supported by the Wales Institute of Cognitive Neuroscience (grant number WBS006) and the Biotechnology and Biological Sciences Research Council (BBSRC) UK (grant BB/G021538). The funders had no role in designing or interpreting the experiment.

## Conflict of interest

SL, MJ, and LS have no competing interests. DH has had consultancies with, been a clinical trialist for, been a speaker at symposia for, or received support to attend meetings from Astra-Zeneca, Boots-Knoll, Eli Lilly, Janssen-Cilag, Lorex-Synthelabo, Lundbeck, Organon, Pharmacia and Upjohn, Pierre-Fabre, Pfizer, Rhone-Poulenc Rorer, Roche, SmithKline Beecham, and Solvay-Duphar. He has been expert witness for the plaintiff in 15 legal actions involving SSRIs and one patent case involving an antipsychotic. DL has received consulting honoraria from Actelion Pharmaceuticals Ltd.

## Figures and Tables

**Table 1:** Demographic and clinical data for both clinical groups and control participants.

	Non-melancholic depression		Melancholia		Controls Melancholia	
	N/mean	SD	N/mean	SD	N/mean	SD
Participants	20		20		20	
NART	113.05	8.98	111.10	10.79	113.85	9.66
Age	42.90	11.16	50.80	10.44	48.50	13.76
Male/female	14/6		10/10		11/9	
Right/left-handed	19/1		19/1		17/3	
HDRS-21	20.85	7.24	25.05	7.49		
Illness duration [months]	174.30	106.09	205.90	177.65		
Antidepressants	TCA: 1^⁎^ SSRI/SSNRI: 12^⁎⁎^ Other: 3 No medication: 4 ^⁎^+ Lithium ^⁎⁎^1 + lithium and 1 + atypical antipsychotic		TCA: 7^⁎^ SSRI/SSNRI: 10^⁎⁎^ Other: 1 No medication: 2 ^⁎^2 + atypical antipsychotic ^⁎⁎^2 + atypical AP 1 + lithium 1 + valproate			

**Table 2:** Criteria for melancholic features specifier (according to DSM-IV) (partly quoted verbatim).

Group A	(1) loss of pleasure in all, or almost all, activities
	(2) lack of reactivity to usually pleasurable stimuli (does not feel much better, even temporarily, when something good happens)
Group B	(1) distinct quality of depressed mood (i.e. the depressed mood is experienced as distinctly different from the kind of feeling experienced after the death of a loved one)
	(2) depression regularly worse in the morning
	(3) early morning awakening (at least 2 h before usual time of awakening)
	(4) marked psychomotor retardation or agitation
	(5) significant agitation or weight loss
	(6) excessive or inappropriate guilt

**Table 3:** D′ (d prime) performance values (and SE in parentheses) of the different groups across emotional categories on the face working memory task.

	Non-melancholic depression	Melancholia	Controls
Angry	3.32 (0.23)	2.91 (0.31)	3.88 (0.17)
Happy	2.91 (0.27)	2.36 (0.19)	3.66 (0.23)
Neutral	2.93 (0.27)	2.65 (0.22)	3.48 (0.21)
Sad	3.02 (0.28)	3.45 (0.22)	3.33 (0.22)
Fearful	2.87 (0.30)	2.23 (0.33)	2.91 (0.19)

**Table 4:** Accuracy (and SE in parentheses) of the different groups for emotion recognition.

	Non-melancholic depression	Melancholia	Controls
Angry	0.71 (0.04)	0.65 (0.06)	0.82 (0.05)
Happy	1.00 (0.00)	1.00 (0.00)	0.99 (0.01)
Neutral	0.70 (0.05)	0.73 (0.07)	0.74 (0.06)
Sad	0.78 (0.04)	0.72 (0.05)	0.87 (0.03)
Fearful	0.76 (0.04)	0.63 (0.06)	0.65 (0.06)
Surprise	0.90 (0.03)	0.91 (0.03)	0.90 (0.03)
Disgust	0.79 (0.06)	0.80 (0.04)	0.87 (0.04)

**Table 5:** SAM ratings (and SE in parentheses) of the different groups for arousal.

	Non-melancholic depression	Melancholia	Controls
Angry	0.89 (0.26)	0.97 (0.25)	1.03 (0.18)
Happy	0.28 (0.24)	− 0.32 (0.40)	0.16 (0.29)
Neutral	− 0.17 (0.23)	− 0.37 (0.17)	− 0.84 (0.30)
Sad	0.22 (0.14)	0.45 (0.26)	0.52 (0.12)
Fearful	1.03 (0.23)	0.90 (0.30)	1.01 (0.22)

**Table 6:** SAM ratings (and SE in parentheses) of the different groups for face valence.

	Non-melancholic depression	Melancholia	Controls
Angry	− 1.15 (0.23)	− 2.10 (0.27)	− 1.44 (0.20)
Happy	1.58 (0.28)	2.38 (0.32)	1.75 (0.28)
Neutral	0.14 (0.13)	0.35 (0.20)	0.35 (0.16)
Sad	− 0.97 (0.17)	− 1.41 (0.28)	− 1.00 (0.18)
Fearful	− 1.12 (0.29)	− 1.83 (0.32)	− 1.46 (0.22)
